# Prevalence and clinical outcomes of *Plasmodium falciparum* and intestinal parasitic infections among children in Kiryandongo refugee camp, mid-Western Uganda: a cross sectional study

**DOI:** 10.1186/s12879-019-3939-x

**Published:** 2019-04-01

**Authors:** Paul Oboth, Yahaya Gavamukulya, Banson John Barugahare

**Affiliations:** 1 Department of Community and Public Health, Faculty of Health Sciences, Busitema University, P.O. Box, 1460, Mbale, Uganda; 2 Department of Biochemistry and Molecular Biology, Faculty of Health Sciences, Busitema University, P.O. Box, 1460, Mbale, Uganda; 3 Department of Biology, Faculty of Science and Education, Busitema University, Tororo, Uganda

**Keywords:** Malaria, Intestinal parasites, Intestinal parasitic infections, Coinfection, Anaemia, Children, Prevalence, Mid-Western Uganda

## Abstract

**Background:**

The prevalence of *Plasmodium falciparum* and Intestinal Parasitic Infections (IPIs) - with the corresponding pathogenesis among children remain uncertain. This study aimed at determining the prevalence and the outcomes (including anaemia) of the respective infections and co-infections. Anaemia is a condition in which the number of red blood cells transporting oxygen to the various body parts is not sufficient to meet the needs of the body.

**Methods:**

This was a cross sectional study conducted among 476-refugee camp school children. Kato-Katz technique was used to screen stool samples for intestinal parasites. Microscopy was used for malaria testing while the portable Haemoglobin (Hb) calorimeter was used to measure haemoglobin concentration.

**Results:**

The overall prevalence of the mixed infections was 63.03%. *Plasmodium falciparum* was most prevalent of the single infections 262(55.04%) followed by *Taenia spp.* 14 (2.9%), *Schistosoma mansoni* 12(2.5%), *Giardia lamblia* 7 (2.9%), *Trichuris trichiura* 2(0.4%), Hookworm 2(0.4%) and *Strongyloides stercoralis* 1(0.2%). The odds of developing simple or uncomplicated malaria infection or anaemia was 14 times higher in individuals with dual co-infection with *Plasmodium falciparum + Taenia sp.* compared to single parasitic infection (Odds = 14.13, *P* = 0.019). Co-infection with *Plasmodium falciparum + Taenia spp,* was a strong predictor of Malaria and anaemia.

**Conclusion:**

This study shows that *Plasmodium falciparum* and *Taenia spp.* co-infections is a stronger predictor of malaria and anaemia. The prevalence of malaria and anaemia remains higher than the other regions in Uganda outside restricted settlements. The findings of this study underline the need for pragmatic intervention programmes to reduce burden of the co-infections in the study area and similar settlements.

**Electronic supplementary material:**

The online version of this article (10.1186/s12879-019-3939-x) contains supplementary material, which is available to authorized users.

## Background

Uganda currently hosts the highest number of refugees in the country’s history and also continues to receive simultaneous emergency influxes from South Sudan, Democratic Republic of Congo (DRC) and Burundi. The Kiryandongo refugee camp has been one of largest camps created to resettle the increasing number of Sudanese refugees in Uganda which has exceeded one million, and the daily arrival rate remains high [1, 2]. Malaria and IPIs are diseases of public health importance particularly among children in Africa. In 2015, Sub-Saharan Africa had 88% malaria cases, 90% of which were fatal [3]. According to Walldorf and others, “School-age children represent an underappreciated reservoir of malaria infection and have less exposure to antimalarial interventions” [4].

Several studies have had limited contribution of intestinal metazoan and protozoa co-infections with malaria in children [5]. Similarly, the interaction between malaria and the different intestinal parasites whenever co-infection is present is inadequately reported.

The most common diseases in the Kiryandongo refugee camp have been previously reported and remain as malaria, upper respiratory tract infections and watery diarrhoea [6]. No study had been performed in a Ugandan refugee resettlement Camp to determine the interaction between malaria and IPIs among children. This study was designed to determine the prevalence of parasitic infections and the respective disease characteristics among the children in the refugee camp.

## Methods

### Study area

**Fig. 1.**
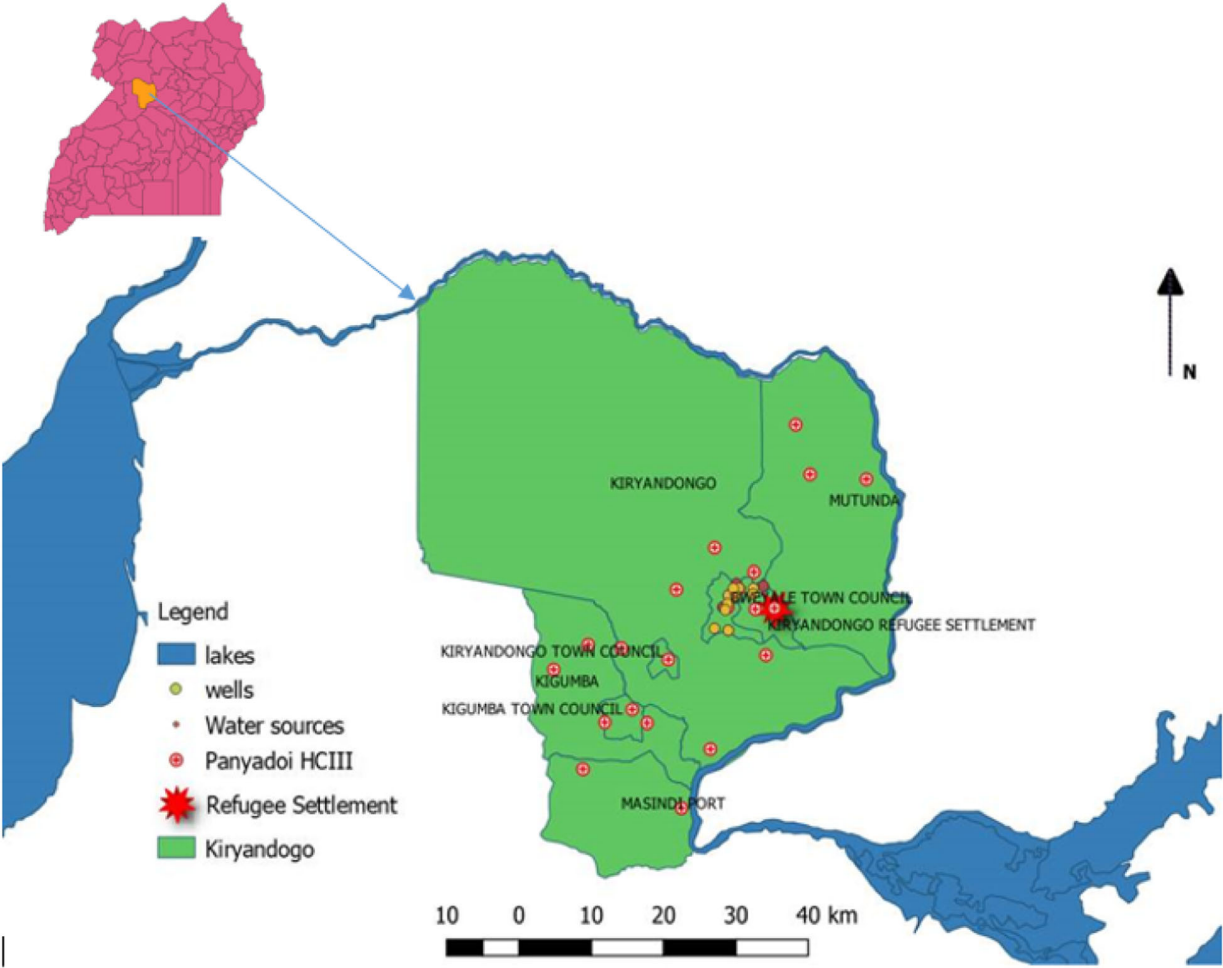
Map of Uganda showing location of Kiryandongo District and the resettlement camp[7].

This study was carried out from October 3, 2017 to May 10, 2018, at Kiryandongo refugee settlement camp in Kiryandongo district, Mid-Western region of Uganda, as shown in Fig. 1 [7]. The Camp is about 255.47 km along Kampala-Gulu road and is surrounded by Lake Albert and Kyoga. The population of Kiryandongo is 280,000 people according to 2014 census and the refugee camp has 52, 613 refugees [8]. The study site was chosen because it falls into a stable malaria transmission zone. Similarly, at 64.9%, the prevalence of anaemia in children aged 6–59 months indicates an increasing trend in Kiryandongo.

### Target population

The primary target population were divided into two groups aged < 5 and 5–14 years as per the local Uganda Ministry of health guidelines. The secondary study population were the care takers/guardians respectively residing within the refugee resettlement camp.

### Inclusion and exclusion criteria

The study included all children who provided both parental consent and assent in addition to providing blood and stool samples. Children below 3 years and above 14 years were excluded from the study.

### Study design

An analytical cross-sectional study design was used to investigate the relationship between *Plasmodium falciparum* and intestinal parasitic co-infection of children. Eligible children aged 3–14 years were recruited and screened for malaria and intestinal parasitic infection. Parents/guardians were interviewed to provide socio-demographic information related to sanitary and malaria prevention practices.

### Sample size calculations

Sample size was determined using the formula for estimating single proportion at the level of 95% confidence interval [Zα/2 = 1.96] by taking the proportion of metazoa and protozoa co-infection prevalence in the study area. However, since the prevalence of co-infection is unknown, was used 0.50 to have the largest sample size. The expected margin of error was taken to be 5% (d = 0.05). Using the formula, n = Z^2^ x P x (1-P)/d^2^. A minimum of 437 samples (n) was estimated as shown: n = Z^2^ P (1-P)/d^2^ [9]**.** Where, n-sample size, P- average prevalence, Z^2^- value at 95% CI, from table, d – expected marginal error. n = (1.96)^2^ × 0.5x (1–0.50) / (0.05)^2^ = 437. Therefore, once the minimum sample was obtained, a contingency of 8% was added for the sample size to reach 476.

### Sampling strategy

Random sampling of children visiting the Panyadoli Health Centre III (HC III) based on the selection criteria was used to obtain blood film and stool sample by the law of voluntarism. Using roll numbers, random numbers were generated using Microsoft Excel 2013. This technique provided each participant with an equal chance of participating in the study. Along with sample collection, face-to-face interview of parents/guardians was conducted using a questionnaire constituting socio-demographic information. The data collection form has been included as Additional file 1.

### Data collection

A researcher developed structured questionnaire based on the objectives of the study, conceptual framework and guided by the literature review was used to collect data from the respondents. The data collection tool with the questionnaire has been included as Additional file 1.

### Laboratory methods

The choice of diagnostic techniques was dependent on available equipment, reagents, experience of the technician and considerations of cost. The techniques used in this study for analysis of stool and blood samples included malaria microscopic examinations using Giemsa Staining technique, intestinal parasitic detection using Kato Katz and Calorimetric Hb estimation. Microscopy was used for detecting malaria because it is a gold standard putting in mind the limitation of rapid diagnostic kits (RDT) since it detects malaria antigen 4 weeks even after treatment and does not allow differentiation of malaria species.

### Data management

Completed questionnaires were serialized and coded. Data was entered and cross checked for errors using Microsoft Excel 2013 before running the analysis.

### Data analysis

The preliminary data was entered into Microsoft Excel 2013 and exported into STATA 14. Descriptive statistics was done to analyse the background characteristics of participants and the factors associated with *Plasmodium falciparum* infection, intestinal parasitic infection and co-infection. Chi-square test and Fishers Exact test was used to determine the association between the factors associated with *Plasmodium falciparum* and intestinal parasites. Statistical significance of the differences in the prevalence of single and co-infection with each factor was determined via Chi-square analysis and Fishers Exact test. Multivariate logistic regression analysis was used to predict the extent of the association with the different infection status as the dependent variable. The study was able to control for confounding factors during analysis by adjusting for age, sex and nutritional status, while the association between helminth and Plasmodium infections, and related outcomes was evaluated.

## Results

### Table 1: Descriptive demographic characteristics of the study population (*N* = 476)

Table 1 above shows the socio-demographic profile of the 476 participants. Most of the study participants were refugees from the South Sudan, Uganda, DRC, Rwanda and Burundi respectively. Of the 476 participants, 364 (83.3%) wash their hands every time after using the toilet and only 73 (16.4%) participants do not wash their hands after using the toilet. 471 (98.9%) wash their hands before eating; and those who do not wash their hands before eating were 6. Most of the participant use borehole 267 (56.1%) and tap water 201 (42.3) as main sources of drinking water. 432 (90.8%) of the participants had previously been infected with malaria. 454 (95.4%) of the children had ever slept under a mosquito net.

**Table 1:** Descriptive demographic characteristics of the study population (*N* = 476)

Characteristics	Frequencies (%)
Country of Origin	
Uganda	37(7.8)
SSD	435(91.4)
Rwanda	1(0.21)
Congo	2(0.42)
Burundi	1(0.21)
Length of stay	
< 12 Months	103(21.6)
12-24 Months	128(26.9)
> 24 months	210(44.1)
Unknown	35(7.4)
Level of Education	
Primary	215(45.2)
Secondary	61(12.8)
Tertiary	18(3.78)
Unknown	175(38.2)
Wash hands before eating	
No	05(1.05)
Yes	471(98.9)
If yes	
Water Only	98(20.8)
Water and Soap	373(79.2)
Source of drinking water	
Tap water	201(42.3)
Borehole	267(56.09)
Stream	2(0.42)
Protected well	3(0.63)
Unprotected	3(0.63)
Ever diagnosed with malaria	
No	15(3.15)
Yes	432(90.8)
Don’t know	29(6.1)
Child ever slept under mosquito net	
No	22(4.6)
Yes	454(95.4)
Source of LLINs	
Gov’t	50(10.6)
NGO	251(52.7)
Don’t Know	175(36.7)
Washing hands after using toilet	
No	73(16.4)
Yes	364(83.3)

Nutritional status assessment using mid upper arm circumference (MUAC), showed no evidence of malnutrition of 100% of children who had median MAUC of 18.8 cm. Out of the 476 children examined, 300 (63.03%) had a mono-infection, 28(5.88%) were co-infected with malaria and IPIs whereas 148 (31.09%) were not, as shown in Table 2 above. It is worth noting that the overall prevalence of parasitic infections in the study population was 63.03%, of which *Plasmodium falciparum* accounted for the highest prevalence 262(55.04%).

**Table 2:** Descriptive study related factors of the study population (N = 476)

Study related factor	Frequencies (%)
Anti-Helminths Medication	
No	117(24.6)
Yes	255(53.6)
Didn’t Know	104(21.9)
MUAC (cm) median	
< 11.5	0 (0.0)
> 11.5	18.8(100)
Haemoglobin(g/dl) median	
< 11.5	11.41(16.7)
> 11.5	13.6 (83.3)
Parasitic Infection	
Single Infection	300 (63.03)
Double-Infection	28 (5.88)
No infection	148 (31.09)

From table 3, the most infected age group by *Plasmodium falciparum* was (5–14 years) with the prevalence of 55.7%. The younger age group (< 5 years) had a prevalence of 52.7% and the difference was statistically significant (*P* = 0.000). A total of 256 (53.8%) males and 220 (46.2%) females were enrolled in the study. Overall, 72.27% (159/220) of the females were slightly more infected with all the intestinal helminths as compared to 55.1% (141/256) of the males but this and the difference was not statistically significant *(P* > 0.05).

**Table 3:** Prevalence of *Plasmodium falciparum* and intestinal parasitic infection, stratified by age and sex

	Parasite species							
	N	*Pf*	*TT*	HW	*GL*	*T.sp*	*SS*	*SM*
Age								
< 5	74	39(52.7)	2(2.7)	0(0)	1(2.5)	4(10.25)	0(0.0)	1(2.5)
5–14	402	223(55.7)	0(0.00)	2(0.89)	6(2.6)	10(4.48)	1(0.45)	11(4.9)
*P*-Value		0.000*	**0.992**	**0.993**	**0.989**	**0.993**	**0.992**	**0.964**
Male	256	123(25.8)	0	0	3(2.15	7(5.03)	1(0.72)	7(5.03)
Female	220	139(29.2)	2(0.91)	2(1.61)	4(3.25	7(5.69)	0(0.0)	5(4.06)
*P-*Value		**0.323**	**0.992**	**0.993**	**0.744**	**0.754**	**0.991**	**0.117**
Totals	**476**	**262**	**2**	**2**	**7**	**14**	**1**	**12**

*Key: Pf = P. falciparum, T.sp = Taenia spp., GL = Giardia lamblia, SS=Strongyloides stercoralis, TT = Trichuris trichiura, SM = Schistosoma mansoni and HW=Hookworms*

As shown in table 4 above, the most prominent co-infection combinations were of *Plasmodium falciparum* + Taenia spp. 5 (1.05%), *Plasmodium falciparum* + *Giardia lamblia* 2 (0.42%) and *Plasmodium falciparum* + *Schistosoma mansoni* 1(0.21%).

**Table 4:** Logistic regression analysis of interaction of *Plasmodium falciparum* and intestinal parasitic co-infections on malaria in the study population

Predictor	Positive n (%)	Odds	*P-*value
*Pf + GL*	2(0.42)	1.674	0.676
*Pf + TT*	1(0.21)	1.00	0.991
*Pf + SM*	1(0.21)	2.25	0.378
*Pf +* HW	1(0.21)	1.00	0.991
*Pf + T.sp*	5(1.05)	14.13	0.019*
*Pf + SS*	1(0.21)	1.00	0.991

*Key: Pf = P. falciparum, T.sp = Taenia sp., GL = Giardia lamblia, SS=Strongyloides stercoralis, TT = Trichuris trichiura, SM = Schistosoma mansoni and HW=Hookworms*

The results above further indicate that the odds of developing simple or uncomplicated malaria infection was 14 times higher in individuals double infected with *Plasmodium falciparum + Taenia spp.* compared to single parasitic infection (Odds = 14.13, *P* = 0.019)**.**

From table 5 above, the occurrence of co-infection of *Plasmodium falciparum + Taenia spp.* (*P* = 0.019, Odds = 14.13) showed a strong predictor of anaemia in the study population. No single infection was a strong predictor of anaemia.

**Table 5:** Logistic regression analysis predicting occurrence of anaemia in relation to parasitic infections in the study population

Predictor	Positive n (%)	Odds	*P-*value
*PF*	262(56.1)	0.82	0.777
*GL*	7(1.47)	1.00	0.984
*TT*	2(0.43)	2.09	0.992
HW	2(0.43)	2.09	0.992
*SM*	12(2.52)	1.10	0885
*T.spp*	14(2.94)	2.62	0.085
*SS*	1(0.21)	1.00	0.991
*PF+ GL*	6(1.26)	0.66	0.707
*PF + TT*	2(0.43)	1.00	0.992
*PF + SM*	12(2.52)	1.11	0.874
*PF +* HW	2(0.43)	1.00	0.992
*PF + T. spp*	5(1.05)	14.13	0.019*
*PF + SS*	1(0.21)	1.00	0.991

*Key: Pf = P. falciparum, T. spp = Taenia spp., GL = Giardia lamblia, SS=Strongyloides stercoralis, TT = Trichuris trichiura, SM = Schistosoma mansoni and HW=Hookworms*

From table 6 above, it is evident that the highest prevalence of anaemia was among the children with no infection at 24.1%.

**Table 6:** Prevalence of anaemia in health children, mono-infection and co-infection in the study population

Infection status	Positive (n)	Prevalence of Anaemia (%)
*No Infection*	148	24.1
*Mono Infection*	300	21.3
*Co-infection*	28	0.0

## Discussion

More than half of the study population tested positive for one parasite (63.03%). The results of this study are similar to the previous report in Tach Gayint, Ethiopia [10] and in the different settlements of Gabon, Central Africa [11]. Similar studies conducted in Cameroon reported higher prevalence of malaria but lower prevalence of anaemia; 64 and 31% respectively [12]. However, in their report the prevalence of anaemia was attributed to malaria [12]. Higher prevalence of anaemia (56.1%) was observed in this study among children infected with *Plasmodium falciparum* followed by *Taenia spp.* at 2.94%. However, studies conducted in North Western Uganda reported a lower prevalence of anaemia (34.4%) of children aged 1-14 years [13]. This was attributed to study setting and intervention strategies by government of Uganda. A higher prevalence of anaemia was previously reported among school children (age 6–14 years) in two Ugandan studies [14, 15].

The current finding of the higher prevalence of malaria and anaemia, is comparable to the previous report by Green et al.*,* 2011 among children living along shores of Lake Albert and in the Islands of Lake Victoria [16]. It is necessarily so, because, in this study the most prevalent helminth was *Taenia spp.* (2.9%). Therefore, the current finding suggests that the co-infection of *Plasmodium falciparum* and *Taenia spp.* is a stronger predictor of anaemia. These results differ from previous reports where the prevalence of the co-infection of 22% [17]. This is perhaps because the malaria prevalence of 77% was higher than of this study conducted in a refugee settlement camp.

The high prevalence of *Plasmodium falciparum* reported in this study is four times higher than previously reported Mid-Western region of Uganda [18]. These discrepancies could be due to the fact that the present study was based in a refugee setting in which febrile children were enrolled, compared to the other studies. Other regional prevalence of 39.7% were reported in East Central, 33.8% in North East, 27.4% West Nile, 20.4% Central 2, 15.3% Mid Northern, 14.1% Mid-Eastern, 10.7% Central-1, 4.2% South Western and Kampala 0.4% among children under the age of five years were reported in Uganda [18]. The difference in the finding could be attributed to the study design, method used, age group and sample size. The study by Roberts and Matthews used data collected from the 2014 Malaria Indicator Survey conducted in Uganda for children under 5 years and the sample size was 10 times (*N* = 4939) higher compared to our sample size.

In this study, the prevalence of intestinal parasitic infection (IPIs) was 7.9%, which is lower compared to the 34.7% reported in communities around Dschang, in the West Region of Cameroon [19], 36.5% in Bushenyi district, western Uganda [20], 22.7% in Thailand [21] and 34.2% in Ethiopia [22]. These discrepancies could be attributed to the geographical differences, regular deworming campaigns by the Ministry of Health, Uganda, sanitation practices and the study sample size. This study reported 53.6% of the study population had taken anti-helminthiasis medication, 99.2% of the sources of drinking water safe and 83.3 and 98.9% children washed their hands after using a toilet and before eating respectively, which may account for the lower prevalence of IPIs.

It is important to note that low socioeconomic status, poor living conditions, insufficient knowledge and practice of correct sanitation, and limited access to safe water are known risk factors for IPIs, are also common in populations from these settlements. This argument is consistent with reports from Ethiopia (70.8% in rural area versus 5.2% in a urban city) and Rwanda (54.5% in a remote area versus 20% in a peri urban area) [11], but contrast with our findings, where most of the children had received long lasting insecticide treated mosquito nets, had safe drinking water and practice good personal hygiene and thus the observed general lower prevalence of intestinal protozoal and helminth infections. Previous studies have shown that intestinal parasitic infections were more prevalent in children aged 5–14 years and attributed to differences in exposure levels in children as they grow [23].

The prevalence of intestinal protozoa was higher than that of intestinal metazoa (1.5 vs. 0.8%), which is in contrast with some studies but in agreement with others due to the urbanization, the public investments in basic sanitation, and improvement of general living conditions, and the accessibility to health services [19, 22, 24]. The observed low prevalence of soil transmitted helminths (STHs) and intestinal protozoa could be attributed to the clean habits of washing hands before eating and after visiting the toilet among the children. This is contrary to the previous reports of higher prevalence of STHs observed among the school children due to poor hygiene practices [25].

A higher prevalence of 13.15 and 9.6% *Taenia spp.* were reported in studies conducted in Ghana [26] and Nigeria [27] respectively. However, this study reported a low prevalence of 2.9% *Taenia spp.,* which is similar to reports previously published across Africa demonstrating taeniasis prevalence ranging from 0 to 8.7%, although these studies do not use a standardized diagnostic protocol [28]. This is in agreement with study conducted in Western Kenya which reported a similarly a very low prevalence of 0.02%, which lies within the previously reported range [29]. This supports the hypothesis that spatial heterogeneity in the distribution of infections may be influenced by environmental conditions, highlighting the interplay between socio-economic, behavioural and environmental factors in *Taenia spp*. infection risk in humans.

In this study, individuals infected with *Plasmodium falciparum* were significantly more likely to harbor *Taenia spp.* 5 (1.05%) and *Schistosoma mansoni* 1(0.21%) compared to individuals without malaria. The most prominent co-infection combinations were of *Plasmodium falciparum + Taenia spp.* 5 (1.05%), *Plasmodium falciparum + Giardia lamblia* 2 (0.42%) and *Plasmodium falciparum + Schistosoma mansoni* 1(0.21%). The factors that supported the co-infections in this study include humidity, surrounding water bodies of Lake Kyoga and Albert, bushes and poor sanitary disposal. The co-infection of *Schistosoma mansoni* and *Plasmodium falciparum* observed in this study has been previously observed [30]. The findings of this study are similar to the previous report from Uganda [31].

The current study shows that the odds of developing malaria and anaemia was 14 times higher in individuals co-infected with *Plasmodium falciparum + Taenia spp.* compared with single parasitic infection. The co-infection of *Plasmodium falciparum + Taenia spp.* showed a stronger prediction of malaria and anaemia (Odds = 14.13, *P* = 0.019). The reason for this co-infection leading to malaria is due to the established immunological antagonism of Th1 and Th2 for *Plasmodium falciparum* and *Taenia* control as has been reported previously in a similar study with other helminthes. *Plasmodium falciparum* and *Taenia* infections are established risk factors of anaemia. Therefore, this co-infection is expected for the observed prevalence of anaemia. Helminthiasis has been shown to significantly contribute to the problem of anaemia in studies conducted in Rwanda, Kenya and Uganda [32–34].

In this study, we did not find statistical relationship between single helminthes infection and anaemia (*P* > 0.05) possibly due to our sample size and the extremely low prevalence. This finding agrees with studies conducted in Uganda around the lake side and island communities of lake Victoria that reported declined prevalence of *Schistosomiasis* from 42.4 to 17.9% in 2005, while hookworm prevalence reduced from 50.9 to 10.7%, *Ascariasis* and *Trichuriasis* from 2.8 and 2.2% in 2003, to very undetectable levels in 2005 [35].

## Conclusion

This is the first study conducted in a refugee settlement camp in Uganda, assessing the prevalence and disease characteristics of *Plasmodium falciparum*, intestinal metazoa and protozoa infections. Our findings show that malaria, intestinal protozoa and helminthes were more prevalent in children aged 3–14 years. Multiple infections with malaria, intestinal protozoa and helminths were common. However, the only significant association observed was between *Plasmodium falciparum* and Taenia spp. This co-infection constitutes an important risk to anaemia and malaria. Secondly, the co-infection impairs the immunological function of the individuals leading to other infections, as established elsewhere. The prevalence of malaria and anaemia remains higher than the other regions in Uganda outside restricted settlements. The findings of this study underline the need for pragmatic intervention programmes to reduce burden of the co-infections in the study area and similar settlements.

## Additional files

Additional file 1 The developed data collection form which contains questionnaire and the associated data collection criterion used in the study. (DOCX 17 kb)

## Abbreviations

GL: *Giardia lamblia*
Hb: Haemoglobin
HC III: Health Centre III
HW: Hookworms
IPIs: Intestinal Parasitic Infections
MUAC: Mid Upper Arm Circumference
Pf: *Plasmodium falciparum*
SM: *Schistosoma mansoni*
SS: *Strongyloides stercoralis*
STHs: Soil Transmitted Helminths
T. spp: Taenia spp.
TT: Trichuris trichiura
UNHCR: United Nations High Commission for Refugees
