# Supplementary material for: Prevalence and clinical outcomes of Plasmodium falciparum and intestinal parasitic infections among children in Kiryandongo refugee camp, mid-Western Uganda: a cross sectional study
Source: BMC Infect Dis. 2019 Apr 1;19:295. doi: 10.1186/s12879-019-3939-x (PMC6444856; doi:10.1186/s12879-019-3939-x)
Supplement: Supplementary file 1 — The developed data collection form which contains questionnaire and the associated data collection criterion used in the study. (DOCX 17 kb) [file 12879_2019_3939_MOESM1_ESM.docx]

## Data collection Form

**General information:** Date of data collection: |______|_______|______|

Data collector code: ………………Respondent Unique N0…………………………………

1. **Socio-demographic Information**

| **No.** | **Question** | **Response** |
| --- | --- | --- |
| 1 | Child class: 0=Nursery, 1= primary >0, 2=Out of school, 3=others | \|__________\| |
| 2 | Child sex: 1=Male; 2=Female | \|__________\| |
| 3 | Child age (years) | \|__________\| |
| 4 | Country of origin | \|__________\| |
| 5 | Length of stay (Months) | \|__________\| |
| **6** | What is the Parent/ guardian level of education?  1=Primary, 2=Secondary, 3=Tertiary level, 4=I don’t know | \|___________\| |

1. **Sanitary related Information**

| **7** | Do you wash hands every time after using a toilet? 0=No, 1=Yes | \|___________\| |
| --- | --- | --- |
| **8** | Do you normally wash hands before eating? 0=No, 1=Yes | \|___________\| |
| **9** | If Yes: 1=water only, 2= water and soap | \|___________\| |
| **10** | What is the source of drinking water? 1=Tap water, 2=Borehole water, 3=Stream water, 4=Protected well, 5=Unprotected well | \|___________\| |

1. **Malaria and intestinal helminth related factors**

|  | **Malaria related factors** | |
| --- | --- | --- |
| 11 | Has the child ever suffered/diagnosed with malaria before? 0=No, 1=Yes, 2= Don’t know/N/A | \|___________\| |
| 12 | Has the child ever received anti-malaria medication? 0=No, 1=Yes, 2= Don’t know/N/A | \|___________\| |
| 13 | Do you use sleep in mosquito net (ITNs/LLINs) 0=No, 1=Yes | \|___________\| |
| 14 | Who supplied you the ITNs/LLINs 1=Gov’t, 2=NGOs | \|___________\| |
|  | **Intestinal parasitic related factors** | |
| 15 | Has the child ever suffered/ diagnosed with intestinal parasitic infection before? 0=No, 1=Yes, 2= Don’t know/N/A | \|___________\| |
| 16 | Has the child ever received anti-helminth medication? 0=No, 1=Yes, 2= Don’t know/N/A | \|___________\| |

1. **Laboratory examination form/findings**

|  | **Nutritional Status** | |
| --- | --- | --- |
| 17 | Mid-upper arm circumference (MAUC) | \|___________\| |
|  | **Analysis of Blood sample** | |
| 18 | Haemoglobin Level (g/L) | \|___________\| |
| 19 | Malaria present: 0=No, 1=Yes | \|___________\| |
| 20 | Malaria parasitaemia (No./2000WBC) | \|___________\| |
| 21 | Malaria status: 1=uncomplicated, 2 complicated/severe | \|___________\| |
|  | **Stool analysis** |  |
| 22 | Helminth present: 0=No, 1=Yes | \|___________\| |
| 23 | Hook worm egg count | \|___________\| |
| 24 | Trichuris Trichiura egg count | \|___________\| |
| 25 | Ascaris lumbricoid egg count | \|___________\| |
| 26 | Giardia cyst/trophozoite count | \|___________\| |
| 27 | E.histolytica cyst/trophozoite count | \|___________\| |
| 28 | Other spp specify | \|___________\| |
